# Interventions supporting cognitive recovery after surgery: a scoping review

**DOI:** 10.1186/s12912-026-04614-y

**Published:** 2026-04-03

**Authors:** Amina Guenna Holmgren, Ulrica Nilsson, Jeanette Eckerblad, Markus Saarijärvi, Agnieszka Kedzierska, Lina Bergman, Carolin Nymark

**Affiliations:** 1 Department of Neurobiology, Care Sciences and Society, Karolinska Institutet, Alfred Nobels Allé 23, C3 | 141 83, Huddinge, Sweden; 2 Department of Cardiology, Danderyd Hospital, Stockholm, Sweden; 3 Gothenburg Centre for Person-Centred Care (GPCC), University of Gothenburg, Gothenburg, Sweden

**Keywords:** Cognitive complications, Cognitive training, Multimodal interventions, Physical activity, Postoperative, Scoping review

## Abstract

**Background:**

The consequences of postoperative cognitive decline have a significant impact on the lives of those affected. These individuals often experience cognitive and emotional difficulties, including disorientation, forgetfulness, fatigue, and slowed processing, which can reduce independence and overall autonomy. This necessitates identifying effective interventions to support postoperative cognitive recovery that are suitable for implementation within the healthcare system.

**Aim:**

To summarize the literature on interventions that support postoperative cognitive recovery following hospital discharge.

**Methods:**

A scoping review was conducted using an iterative approach on 2 February 2025, with two updated searches performed on 16 April 2025 and 17 October 2025. Searches were done in three databases (Medline via Ovid, PsycInfo and CINAHL). Eligibility criteria were limited to quantitative studies of participants (≥ 18 years) undergoing surgery, focusing on interventions. Eligible studies had to report at least one cognitive outcome measure. Reporting adhered to the Preferred Reporting Items for Systematic reviews and Meta-Analyses extension for Scoping Reviews (PRISMA-ScR).

**Results:**

The scoping review included 21 studies (*n* = 2563 participants) evaluating three types of interventions: cognitive training (*n* = 12), multimodal interventions (*n* = 7) and physical activity (*n* = 2). The duration and timing of the interventions varied considerably both within and between intervention types. All studies utilised some form of neurocognitive test to assess cognition. Of 12 studies on cognitive training, three reported positive effects, and two of seven multimodal interventions also showed positive results. Among the studies focused on physical activity, one of the two demonstrated a positive effect.

**Conclusions:**

This review indicates that current evidence is insufficient to support interventions that improve postoperative cognitive recovery; the evidence base remains fragmented and lacks strong theoretical grounding. Future research should prioritise standardised outcome measures, robust conceptual frameworks, and large-scale, methodologically rigorous trials to strengthen the evidence base and inform clinical practice.

**Clinical trial number:**

Not applicable.

**Supplementary Information:**

The online version contains supplementary material available at 10.1186/s12912-026-04614-y.

## Introduction

Postoperative cognitive recovery refers to the process through which a patient returns to their normal cognitive functioning after surgery [1]. The course of recovery varies widely among individuals and may involve a range of cognitive challenges associated with postoperative cognitive decline, which in some cases can persist for several years [2, 3]. Following the recommendations of the nomenclature for cognitive change associated with anaesthesia and surgery [2], postoperative delirium (POD) is characterized by a sudden decline in attention and cognitive function occurring within seven days after surgery, while cognitive decline occurring within 30 days after surgery is classified as delayed neurocognitive recovery (dNCR). If this decline continues for up to a year after the procedure, it is classified as postoperative neurocognitive disorder (p-NCD) [2, 4–7]. The occurrence rate of dNCR and p-NCD ranges from 10% to 47%. The cognitive changes associated with dNCR and p-NCD can range from mild to severe and may impact one or more cognitive abilities [4, 5, 7, 8]. The etiology is suggested to be multifactorial, but previous research has demonstrated a clear link between central inflammation caused by surgery and dNCR and p-NCD [3, 9–11], particularly in patients over the age of 65 with pre-existing cognitive disorders, depression, poor functional status and frailty, as well as longer duration of surgeries [12–16].

Patients suffering from dNCR/p-NCD often describe their condition by saying they “haven’t been the same since the surgery” and often attribute it to medications and their side effects [17, 18]. They often experience a range of cognitive challenges, including difficulties with orientation, forgetfulness, fatigue, misplaced belongings, reading difficulties and slower cognitive processing [19, 20]. Emotional responses, such as feelings of sadness, anger outbursts, and anxiety, are also symptoms connected to cognitive decline after surgery. These cognitive symptoms and experiences are significantly correlated with a loss of independence, as they can lead to difficulties in daily activities and depression [19, 20]. This, in turn, diminishes autonomy and personal capability [18–21]. In addition, early cognitive complications, i.e. postoperative delirium, may lead to lasting cognitive challenges. These challenges include difficulty with orientation, forgetfulness, fatigue, frequently misplacing belongings, reading difficulties, slower cognitive processing, and feelings of sadness [22, 23]. This overlap makes it challenging to differentiate between delirium-related cognitive impairment and dNCR/ p-NCD.

Given the significant consequences associated with postoperative neurocognitive decline, identifying effective non-pharmacological interventions (hereafter referred to as ‘interventions’) that are feasible for implementation within healthcare systems is essential. However, current knowledge remains fragmented regarding the effectiveness, timing, and characteristics of such interventions. For example, although strategies such as cognitive training have been investigated, it remains unclear whether their effects differ depending on whether they are delivered preoperatively or postoperatively, and which approaches yield the greatest benefit [24–26]. Furthermore, previous reviews have primarily focused on intervention outcomes and have not comprehensively examined the range of interventions or the theoretical assumptions underlying their design.

Interventions may be based on different theoretical assumptions and target distinct mechanisms, but the extent to which these assumptions inform intervention design and implementation remains unclear. Scoping reviews are particularly appropriate for identifying the types of available evidence, clarifying key concepts, and mapping the characteristics and underlying assumptions of interventions in heterogeneous and evolving fields [27]. This approach is especially relevant when the literature is fragmented and not amenable to quantitative synthesis. Accordingly, a scoping review was considered the most suitable methodology to map the breadth, characteristics, and theoretical assumptions of interventions targeting postoperative neurocognitive outcomes, and to identify knowledge gaps.

### Aim

To summarise the existing literature on interventions supporting postoperative cognitive recovery after discharge from the hospital. This was explored using six research questions:

1. Which patient populations were targeted by the interventions?
2. What type of interventions have been tested?
3. What was the timing and the duration of the intervention?
4. When and how were the cognitive outcomes measured?
5. What were the findings from the interventions?
6. What was the theoretical framework underlying these interventions?

## Methods

A scoping review was conducted. Scoping reviews are valuable for mapping and synthesising existing evidence and can serve as a foundation for guiding future research and implementation efforts. The research process followed an iterative approach based on the methodological framework by the Joanna Briggs Institute [28], The Preferred Reporting Items for Systematic reviews and Meta-Analyses extension for Scoping Reviews was applied (PRISMA-ScR) (Supplementary file 11) [29].

### Search methods

A literature search was performed in the following databases: Medline (Ovid), PsycInfo (EBSCOhost) and CINAHL (EBSCOhost). After the initial search conducted on 2 February 2025, the search was updated on 16 April 2025 by rerunning the searches, adding search terms related to pilot studies and feasibility studies to ensure comprehensive coverage of emerging evidence, and deduplicating against previous results using a combination of the methods described by Bramer [30]and Covidence (www.covidence.org). These additions were made to broaden the scope of eligible study designs and were not based on the initial search results. The original search terms were otherwise unchanged. A final search update was performed on 17 October 2025 to ensure the most recent literature was included.

The search strategy was developed in Medline (Ovid) in collaboration with librarians at the Karolinska Institutet University Library and included search terms related to the study aim (Supplementary file 2). For each search concept Medical Subject Headings (MeSH-terms) and free text terms were identified. The search was then translated, in part using Polyglot Search Translator [31], into the other databases. No language restriction was applied; however, only articles written in English were included. For all database searches, a filter was added to capture randomised controlled trials. All filters were broadened to include as many relevant articles as possible [32, 33]. The search strategies were peer reviewed by another librarian prior to execution.

### Inclusion criteria and exclusion criteria

Eligible studies included controlled non-pharmacological intervention studies conducted among adult participants (≥ 18 years) undergoing surgical procedures. The focus was on interventions delivered before or after surgery that addressed postoperative cognitive outcomes, including dNCR, POCD, p-NCD, and pNCD. Interventions were defined broadly as any planned perioperative non-pharmacological, non-surgical treatment, program, or strategy implemented before or after surgery and intended to influence postoperative cognitive outcomes, either as a primary or secondary endpoint. To be included, studies had to report at least one cognitive outcome measure. Only primary research published in peer-reviewed scientific journals in Swedish or English, with no time restrictions, was considered.

Studies were ineligible if the intervention took place exclusively during surgery, involved pharmaceuticals or surgical procedures, or focused solely on delirium outcomes, i.e., by using screening tools designed to detect delirium, such as the NuDESC or CAM-ICU, and by measuring cognitive outcomes only during the first seven postoperative days.

In addition, studies involving neurosurgical procedures were excluded, as these patients may experience neurological complications that are difficult to distinguish from postoperative cognitive decline.

### Selection of records

Search results were exported to the Covidence systematic review software ™ for processing. After duplicates were removed, five of the authors (first, second, third, sixth, and last author) independently screened the titles and abstracts to determine eligibility. Each article required a vote from at least two reviewers to proceed; any conflicts were resolved by a third reviewer. Throughout the screening process, the first and last authors continuously met to discuss uncertainties, and all authors were regularly informed and involved in resolving disagreements.

**Fig. 1:**
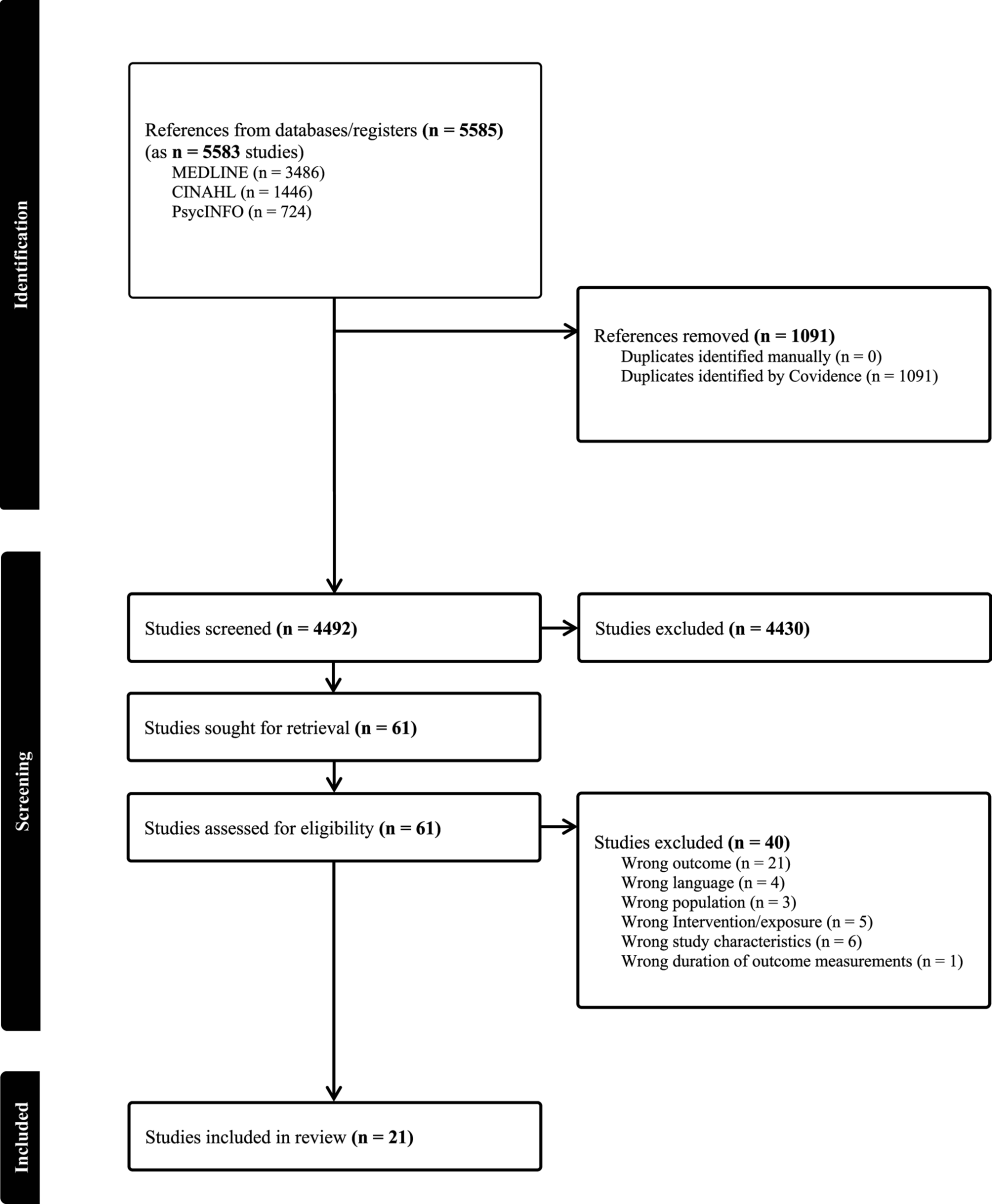
PRISMA flow diagram

Subsequently, two of the authors conducted a full-text review (the first and last author). Each article again required a vote from two reviewers to be included, with any conflicts resolved by the sixth author. In total, 21 studies met the predefined inclusion criteria. A PRISMA flow diagram (Fig. 1) illustrates the search and selection process.

### Data abstraction

A customised data extraction form was developed in Covidence ™ to ensure systematic data collection. The data extraction form captured general study characteristics, including title, authors, year of publication, country, and study setting. In addition, it documented details related to the intervention, including its target population, timing and duration, outcome measures, and underlying theoretical framework. The data extraction form was piloted across three studies by the first and last author to assess consistency, clarity, and usability, resulting in minor refinements. Thereafter, the first and last authors independently reviewed and extracted data from all included studies. Their assessments and extracted data were then compared and discussed to achieve consensus.

### Synthesis

Using the extracted data and guided by the six predefined research questions, the second and last authors selected specific details from each article to include in the results. The results were summarised, compared for similarities and differences, and then narratively synthesised. This process made it possible to identify common features, variations, and gaps in the current evidence base [34]. The second and last author each analysed half of the research questions, then compared their findings and discussed them to reach a consensus. The interpretations were refined through additional discussions among the first, second, and last authors to ensure consistency and transparency. Finally, the results were summarised in text and tables structured around the research questions, with interventions grouped by type to facilitate comparison of cognitive outcomes across categories, and to provide an overview of theoretical foundations, target groups, timing, outcome measures, and main findings.

## Results

### Overview of selected studies

Initially, 5583 studies were identified through a database search. Ultimately, 21 studies met the inclusion criteria, in which one study was published in three separate articles [35–37]. The total sample comprised 2563 participants, including 1334 participants in the intervention group (IG) and 1229 in the control group (CG) (Table 1).

#### Patient populations targeted by the interventions

The number of participants ranged from 6 to 284 in the IG and 4 to 247 in the CG. The most common type of surgery the participants underwent in the included studies was cardiac surgery (*n* = 10), followed by various orthopaedic surgeries (*n* = 6), urological surgery (*n* = 1), lung transplantation (*n* = 1), vascular surgery (*n* = 1), and various elective non-cardiac surgeries (*n* = 3). Participants’ ages ranged between 57 and 84 years in the IG and 57 and 85 years in the CG. The oldest patients were those undergoing orthopaedic surgery, with an age range of 65–84 years in the intervention group and 70–85 years in the control group (Table 1). All studies included both genders, with the proportion of females ranging from 17% [35–38] to 79% [39]. No information on gender was found in three studies [40–42].

**Table 1:** Characteristics of the included studies

Study and country	Type of surgery	Sample size IG/CG	Age (mean) IG/CG	Type of intervention and when	Description of the intervention	Control group
Ajtahed 2019, Iran	CABG surgery	25/47	57.0/57.0	Cognitive training. After surgery	24 computerised cognitive training sessions, combined with routine cardiac rehabilitation, were delivered in 20-minute sessions over 8 weeks. This cognitive training program targeted various cognitive functions, including attention, working memory, and inhibition.	A sham version plus cardiac rehabilitation
Butz, 2022; Butz, 2023a; Butz, 2023b Germany	Cardiac surgery	47/47	71.2/73.0	Cognitive training. After surgery	Paper-and-pencil exercises targeting multi-domain cognitive executive functions began one week after surgery. Sessions lasted 36 min and were held six days a week for three weeks, for a total of 18 sessions.	Usual care
Carbone 2019, Italy	Partial arthroplasty of the knee or total arthroplasty	18/16	69.5/69.7	Cognitive training. Before and after surgery	The recall of audio-recorded words occurred over five individual sessions. The first session took place before surgery, while the fifth session lasted about 90 min. The second, third, and fourth sessions each lasted approximately 30 to 40 min and were completed within a two-week period, with a fixed two-day break between sessions.	Alternative activities.
Cetinkaya 2022, Turkey	Transurethral bladder resection	30/30	69.2/69.6	Cognitive training and telephone support. After surgery	Simple calculations for at least 15 min each day for four weeks, in total 28 sessions, following their surgery. The researchers contact each participant twice a week. During these phone calls, the conversation lasted about five minutes and focused on a specific topic. In the subsequent call, the researchers evaluated whether the participants remembered the previous discussions.	Usual care
Greaves 2023, Australia	Cardiac surgery	18/18	73.8/72.6	Cognitive training. Before and after surgery	Computerised cognitive training using a platform that offered a selection of exercises aimed at specific cognitive domains. Training sessions lasted 45–60 min and were held every other day before surgery. The total number of sessions was not reported. After surgery, the sessions were held three times a week, starting one month after surgery and continuing for a total of 12 weeks, with a total of 24 sessions.	Contacted weekly via phone for a pain assessment
Jiang 2024, China	Cardiac surgery	102/106	65/66	Cognitive training. Before surgery	Computerised cognitive training using a dynamic mobile application focused on cognitive exercises. Participants were instructed to complete 10 h of training, dedicating at least 1 h each day i.e. 10 sessions. They were to engage in the training over two to three sessions, ensuring that each daily session included at least one game from each of the six available cognitive domains.	Usual care
Kulason 2018, Japan	Non-cardio vascular thoracic surgery	6/4	69.0/68.8	Cognitive training. After surgery	Simple calculations and reading aloud. The difficulty level of the arithmetic materials varied. The participants were instructed to complete the arithmetic exercises as quickly and accurately as possible and to stop after 15 min for each training session. The 30-minute intervention was delivered three to five times per week for 12 weeks, resulting in 36–60 sessions.	Usual care
O’Gara 2020, United States	Cardiac surgery	20/20	70.0/69.0	Cognitive training. Before and after surgery	Computerised training via a mobile application with programs designed to enhance cognitive skills. Participants were instructed to train in two 15-minute sessions each day, beginning before surgery at enrollment, (19–38 sessions) and to continue for four weeks after surgery, i.e. 28 sessions, resulting in a total of 47–66 sessions.	Usual care
Ros-Nebot 2024, Spain	Elective non cardiac surgery	46/34	68.3/67.8	Cognitive training. Before and after surgery	Computerised training using a mobile application, including three different games that both stimulate and work on different cognitive processes. Each game lasted 15 min and was conducted daily for 10 days before surgery i.e. 10 sessions.	Usual care
Song 2019, China	Lung transplant	23/23	65.0/66.8	Cognitive training. After surgery	Computerised training included eight exercises: four focused on improving attention and information processing speed, while the other four targeted working memory. Participants completed four exercises, each lasting 10 min, five days a week, for 8 weeks, resulting in a total of 40 sessions.	Usual care
Tarasova 2023, Russia	Cardiac surgery	70/40	65.3/65	Cognitive training. After surgery	Two intervention groups: a postural balance task with mental arithmetic, verbal fluency, and divergent tasks, or a simple visual–motor reaction with mental arithmetic, verbal fluency, and divergent tasks. Start 3 to 4 days after surgery, once daily for 5 to 7 days. Each daily training session should last 20 min, i.e. 5–7 sessions.	Usual care
deTournay-Jette 2012, Canada	CABG surgery	26/18	69.9/70.9	Cognitive training. After surgery	Two intervention groups: one received attention training followed by memory training, while the other received memory training followed by attention training. Each training was composed of four 50-minute sessions, conducted twice per week, starting 1 month after surgery. Between the eighth and tenth weeks, the groups switched training focuses, with the attention training group moving to memory training and the memory training group moving to attention training. End of the intervention after 3 months, resulting in 36 sessions.	Usual care
Liang 2021, Taiwan	Orthopedic surgeries	81/59	70.7/71.9	Multimodal intervention. After surgery during hospitalisation	The intervention, the Hospital Elder Life Program, included four protocols: orientation and communication, early mobilisation to prevent immobilisation, provision of equipment for vision and hearing impairments, and early intervention for volume depletion to prevent dehydration.	Usual care
Olotu 2022, Germany	Cardiac surgery	284/247	71.7/71.9	Multimodal intervention. After surgery during hospitalisation	A practical approach involves installing clocks and calendars in patient rooms and providing newspapers, journals, and books. Support of nutrition and sleep. Ensure patient mobility, by manage central venous lines, gastric tubes, Foley catheters, and surgical drains. Nutrition	Usual care
Shyu 2013, Taiwan	Accidental single-side hip fracture	79/81	77.4/79.0	Multimodal intervention. After surgery during hospitalisation	The interdisciplinary intervention program included geriatric consultation services, a continuous rehabilitation program, and discharge-planning services and lasted until 3 months after discharge. The geriatric assessment/consultation was administered by a geriatrician and geriatric nurses during hospitalisation, before and after surgery, to detect potential medical and functional problems and to decrease delays before surgery. Continuous rehabilitation included inpatient and at-home programs and was delivered by geriatric nurses and physical therapists.	Usual care
Wang 2020, China	Elective surgical procedure with an anticipated LOS longer than 2 days.	152/129	74.2/75.3	Multimodal intervention. After surgery during hospitalisation	The intervention, the Hospital Elder Life Program, consisted of 3 universal protocols and eight targeted protocols. The universal protocols include orientation, therapeutic activities, and early mobilisation. Individualized intervention was subsequently provided daily. To ensure family adherence, family education was provided to help the family members understand the importance of their work.	Usual care
Watne 2014, Norway	Hip fracture (a femoral neck fracture, a trochanteric or a sub-trochanteric fracture)	163/166	84/85	Multimodal intervention. After surgery during hospitalisation	Comprehensive Geriatric Assessment for treatment planning. On the patients’ first day in the ward, all team members—including the geriatrician, nurse, physiotherapist, and occupational therapist—conducted assessments. The team hold daily meetings to coordinate treatment plans and discuss discharge arrangements. Clinical routines were developed through a literature review, insights gained from previous orthogeriatric models. Checklists are provided for each patient to guide the treatment team. These clinical routines encompass medication reviews, early and intensive mobilisation, the optimisation of pre- and postoperative nutrition, and planning for early discharge.	The control group was treated in the orthopedic ward
Mao 2022, China	Carotid revascularization surgery	49/48	73.0/68.0	Multimodal intervention. With nurse-led home visits and telephone support. After surgery	Home visits and telephone support by trained nurses with goals such as increasing positive thoughts and self-perception, reducing psychological pressure, and improving problem-solving strategies. Home visits were performed at weeks 0, 4, 8, and 12 and telephone support at weeks 2, 6, and 10, whereas the control group received all home visits at weeks 0, 2, 4, 6, 8, 10, and 12.	Home visits at week 0, 2, 4, 6, 8, 10, 12, but no telephone support
Shen 2022, China	Total hip arthroplasty	25/25	71.5/74.6	Multimodal intervention. After surgery during hospitalisation	Multimodal interventional procedures conducted by trained nurses during hospitalisation include cognitive, emotional, environmental, educational, nutritional, and sleep interventions.	Usual care
Pengelly 2021, Australia	Cardiac surgery	20/18	70.5/74.1	Two different physical activity interventions. After surgery	The program was an aerobic-based rehabilitation initiative conducted in a community for 12 weeks, with sessions held once a week. It primarily includes light-intensity aerobic exercises, unweighted exercises for both upper and lower limbs, and stretching activities. Each session lasts between 30 and 45 min, totalling 24 sessions.	No control group/usual care.
Trubnikova 2021, Russia	Cardiac surgery	50/53	59.0/58.0	Physical activity. Before surgery	Aerobic physical prehabilitation training once daily for 5–7 days before surgery. The daily training session lasted 40 min.	Usual care

#### Type of interventions

Of these 21 studies, 12 evaluated the effects of cognitive training [35–38, 40, 41, 43–50], with six of these utilising computerised cognitive training (CCT), primarily focusing on exercises intended to improve working memory and attention [38, 43, 46, 48–50]. Among these, one study utilised a mobile application featuring three distinct games designed to stimulate and engage different cognitive processes [49]. Seven of the studies involved various multimodal interventions, which included components such as team-based treatment planning, comprehensive geriatric assessments, orientation to time and space, therapeutic activities, early mobilisation, optimisation of pre- and postoperative nutrition, planning for early discharge support, strategies to promote sleep, home visits, telephone support after discharge, and various approaches aimed at emotional and cognitive improvement during hospitalisation [39, 51–56]. Two interventions focused on physical activity, primarily aerobic exercises [42, 57]. All interventions primarily targeted the patient, except for Wang’s multimodal intervention, which also incorporated the patient’s family [54]. For more details of the interventions, see Table 1.

#### Timing and duration of the intervention

##### Cognitive training

The length of each session ranged from 15 min [44, 48] to a maximum of 90 min [40], and the total number of sessions ranged from 5 [40, 41] to 66 [48]. The frequency also differed, ranging from daily sessions [41, 44, 46, 48, 49] to twice-weekly sessions [45]. Of the 12 interventions targeting cognitive training, two were conducted solely in the preoperative phase [46, 49], three included both preoperative and postoperative training components [38, 40, 48], and in seven, cognitive training was provided postoperatively [35–37, 41, 43–45, 47, 50]. For more details on timing, frequency, duration and number of sessions, see Table 1.

##### Multimodal interventions

The duration of the interventions varied significantly by type and timing, and it was not possible to specify the exact duration due to their individualized, flexible nature, which was adapted to patients’ clinical needs and contextual factors. Therefore, they are not specified with respect to the length of stay or reported follow-up time. Seven studies [39, 51–56] explored multimodal interventions implemented during hospitalisation after surgery. Hospital stay durations ranged from 5.4 [39] to 13.2 days [56]. Additionally, Shyu’s intervention was maintained for three months following discharge [53], and Mao’s intervention involved four home visits and four telephone calls, each with a specified duration depending on the type of support [55]. For more details, see Table 1.

##### Physical activity

In one study, the intervention was conducted during the pre-operative phase, consisting of daily sessions lasting 40 min each for up to seven days, for a total of 280 min [42]. The other study took place weekly over a 12-week period postoperatively, with each session lasting between 30 and 45 min [57]. For more details of the timing and duration of the interventions, see Table 1.

#### The cognitive outcomes

All studies utilised various neurocognitive tests to evaluate cognition. Thirteen studies employed a comprehensive neurocognitive test battery [35–38, 40–43, 45, 47, 49–52, 55], while eight studies [39, 44, 46, 48, 53, 54, 56, 57] used one or two individual cognitive screening tests; the Mini-Mental State Examination; MMSE was the most common (*n* = 5), followed by the Confusion Assessment Method; CAM (*n* = 3) and the Montreal Cognitive Assessment, MoCA (*n* = 2). In two studies, cognition was assessed by a relative or next of kin [37, 51], and in one study, the patient self-evaluated their own cognitive abilities [37]. Baseline cognition was primarily assessed preoperatively, except in four studies [43, 47, 50, 55]. Additionally, one study did not conduct a baseline assessment, instead using the same neurocognitive test battery to evaluate postoperative cognitive function [51]. Post-intervention measurements occurred anywhere from 24 h after surgery [42] to 24 months later [53], with the number of assessments ranging from 1 to 4, with the most common number being 3. For more details, see Table 2.

**Table 2:** Cognitive outcomes and time point of assessment

Study	Neurocognitive test battery	1–2 cognitive tests	Time points for assessment
Ajtahed 2019, Cognitive training	+		Baseline: postoperatively, before the intervention, Post-intervention: week 8 and after 6 months
Butz, 2022; Butz, 2023a; Butz, 2023b Cognitive training	+		Baseline preoperatively Post-intervention: upon discharge from rehabilitation and 3 months after discharge
Carbone 2019, Cognitive training	+		Baseline: preoperatively, before the intervention Post-intervention: postoperatively after the last training session
Cetinkaya 2022, Cognitive training		+	Baseline: preoperatively Post-intervention: postoperative day 1 and 30
Greaves 2023, Cognitive training	+		Baseline preoperatively Post-intervention: at discharge, at 4 and 6 months after the intervention
Jiang 2024, Cognitive training		+	Baseline: preoperatively Post-intervention: postoperative day 7, or at discharge and at 1 month after surgery
Kulason 2018, Cognitive training	+		Baseline postoperatively Post-intervention: 3 months after the intervention
O’Gara 2020, Cognitive training		+	Baseline: Day of enrollment, on the day of surgery, Post-intervention: on the day of discharge and at 1, 3, and 6 months postoperatively
Ros-Nebot 2024, Cognitive training	+		Baseline: Preoperatively Post-intervention: at 30 days postoperatively
Song 2019, Cognitive training	+		Baseline: Before intervention Post-intervention: immediately after the intervention and 12 weeks after the intervention
Tarasova 2023, Cognitive training	+		Baseline: Preoperatively Post-intervention: 11–12 days postoperatively
deTournay-Jette 2012 Cognitive training	+		Baseline: Preoperatively Post-intervention: postoperatively after 3–10 days and 1 and 6 months
Liang 2021, Multimodal intervention		+	Baseline: preoperatively Post-intervention: at 1-, 6-, 12-months after discharge
Olotu 2022 Multimodal intervention	+		Baseline: Preoperatively Post-intervention: postoperatively at 1 week 3 and 12 months, and 12 months after surgery
Shuy 2013, Multimodal intervention		+	Baseline: Preoperatively Post-intervention: at 6, 12, 18, and 24 months after discharge
Wang 2020, Multimodal intervention		+	Baseline: Preoperatively Post-intervention: 1 month after discharge
Watne 2014, Multimodal intervention	+		Baseline: No baseline assessments using the neurocognitive test battery that assessed postoperative cognitive function were conducted. Post-intervention: 4 and 12 months postoperatively
Mao 2021 Multimodal intervention	+		Baseline: Postoperatively, after discharge before the intervention Post-intervention: Postoperatively at 1 and 3 months after the intervention
Shen 2022, Multimodal intervention		+	Baseline: Preoperatively Post-intervention: day 1, 7 and 14 days postoperatively
Pengelly 2021 Physical Activity		+	Baseline: preoperatively Post intervention: 14 weeks and 6 months postoperatively
Trubnikova 2021, Physical Activity	+		Baseline: Preoperatively Post-intervention: 7–10 days postoperatively

Neurocognitive test battery = a structured collection of multiple cognitive tests covering several domains; Cognitive test = one specific measure

#### Findings from the interventions

Postoperative cognitive decline outcomes were categorised as dNCR and p-NCD based on when they were collected, as none of the included studies used the new nomenclature. Eleven out of 21 studies measured dNCR [36, 41, 42, 45, 46, 48, 49, 52, 54, 56]. Four studies reported a positive effect of the intervention on dNCR, including two that evaluated cognitive training. In Butz et al. (2022), improvements were observed at discharge from rehabilitation and again three months later [36], and in Ros-Nebot et al. (2024), effects were noted 30 days after surgery [49]. The other two studies included one assessing a multimodal intervention, which showed an effect at one month post discharge [54], and one evaluating physical activity, which demonstrated benefits 11–12 days postoperatively [42].

The other seven studies measuring dNCR showed no effect of the intervention [41, 45, 46, 48, 52, 56, 57]. Only five of the 21 included studies measured p-NCD [35, 36, 48, 50, 52, 57]. One of them, Butz et al., who tested cognitive training, demonstrated a positive effect at three months after discharge [36] and again at 12 months [35]. In 13 studies examining different aspects of postoperative cognitive function [38–41, 43, 44, 47, 49, 51, 53, 55–57], some outcomes indicated improvements, as presented in Table 3. While the intervention showed a positive impact on cognitive function in certain studies, the control group’s cognitive function remained stable [40, 43, 44, 47]. Additionally, there was one study in which both groups showed improvements [41] and others in which both groups showed a decrease in postoperative cognitive function [39, 53]. In one study, both self-assessed cognitive failure and memory failure were evaluated, along with next-of-kin assessments of cognitive failure. The intervention did not affect these outcomes [37] (Table 3).

The studies also examined secondary outcomes, including delirium (*n* = 6), depression (*n* = 5), daily living activities (*n* = 4), quality of life (*n* = 3), physical function (*n* = 2), fragility (*n* = 2), mortality (*n* = 1), mobilisation (*n* = 1), nutrition (*n* = 1), recovery quality (*n* = 1), biomarkers (*n* = 1), and satisfaction (*n* = 1). The effects on these outcomes varied, with both positive and no effects, as shown in Table 3.

**Table 3:** Results from the interventions

Study	Intervention	Postoperative cognitive decline	Postoperative cognitive function	Other outcomes	Comments
Ajtahed, 2019	Cognitive training		+Working memory -Substation, divided and selected attention	+QoL	No decline in the control group, just improvement of the cognitive function in the intervention group
Butz, 2022; Butz, 2023a; Butz, 2023b	Cognitive training	+dNCR +p-NCD	-Self-assess cognition failure -Next of kin assesses cognitive failure -Self-assessed memory failures	+QoL -Depression	
Carbone, 2019	Cognitive training		+Working memory -Short-term memory -Long-term memory	-Depression	No decrease in cognitive function in the control group, just improvement of working memory in the intervention group
Cetinkaya, 2022	Cognitive training		+Cognitive function	+Depression	No decrease in cognitive function in the control group, just improvement of the cognitive function in the intervention group
Greaves, 2023	Cognitive training		-Global cognition performance	- Delirium	
Jiang, 2024	Cognitive training	-dNCR		+Delirium -Mortality	
Kulason, 2018	Cognitive training		+Improved cognitive performance -Working memory -Substation, divided and selected attention	+QoL +Depression	No decrease in cognitive function in the control group, just improvement of cognitive performance in the intervention group
O’Gara, 2020	Cognitive training	-dNCR -p-NCD		-Delirium	
Ros-Nebot, 2024	Cognitive training	+dNCR	+Objective memory disturbance and failure		
Song, 2019	Cognitive training	-p-NCD			
Tarasova, 2023	Cognitive training	-dNCR	+Psychomotor and executive functions +Attention +Short-term memory		Improved short-term memory and attention were seen in all groups
deTournay-Jette, 2012	Cognitive training	-dNCR			Larger improvement in attention and memory by cognitive training, yet the control group also improved. No statistical significance
Liang, 2021	Multimodal intervention		+Greater preservation of cognitive function	-Delirium -ADL -Depression -Fragility -Nutrition -Muscle strength -Gait speed	Decrease in cognitive function in both groups
Olotu, 2022	Multimodal intervention	-dNCR -p-NCD		-Delirium	Delayed neurocognitive recovery in both groups
Shyu, 2013	Multimodal intervention		-Cognitive function		In both groups, cognitive function decreased
Wang, 2020	Multimodal intervention	+dNCR		+Delirium +Fragility +ADL	
Watne, 2014	Multimodal intervention		-Cognitive function	-ADL	
Mao, 2021	Multimodal intervention		+Cognitive function in 4/6 tests at 3 months -Cognitive function at 1 month		
Shen, 2022	Multimodal intervention	-dNCR	+Cognitive function	+Early mobilitation +Satisfaction	
Pengelly, 2021	Physical activity	-dNCR -p-NCD	+Cognitive function	-Quality of recovery -Physical function -ADL	
Trubnikova, 2021	Physical activity	+dNCR		+S100b -NSE +BDNF	

Abbreviations: dNCR=Delayed neurocognitive recovery i.e. cognitive decline occurs within 30 days post-surgery; p-NCD=Postoperative neurocognitive disorder i.e. the decline continues from 30 days for up to a year after the procedure; QoL= Quality of Life; ADL=Activity of Daily Living; NSE=Neuron-Specific Enolase; BDNF=Brain-Derived Neurotrophic Factor; S100b=Calcium-Binding Protein B

#### Theoretical framework underlying the intervention

##### Cognitive training

Several of the studies investigating cognitive training had no reference to an underlying theory [38, 43–45, 50] and attribute improved performance in memory and attention tasks to the training [37, 40, 43–45]. Some suggested that cognitive training could enhance patterns in brain structure, known as intervention-related alterations in neuroplasticity [36, 45] or increase the cognitive reserve [46, 48, 49]. Other theories suggest that cognitive training may stimulate the frontal cortex, as well as the association areas in the temporal and parietal lobes. This stimulation could result in enhancements in the functioning of these brain regions [47]. When multitasking during cognitive training, it was suggested that this approach activates widespread areas of the brain, particularly the frontal and parietal cortices. These regions play a crucial role in distributing attention during information processing. Additionally, these areas are known to be watershed zones for blood supply, located at the borders between different vascular pools [41].

##### Multimodal intervention

One of the study’s theories suggested that a multimodal intervention aimed to prevent postoperative delirium could help reduce the incidence of p-NCD [51, 52] and maintain or enhance cognitive function after discharge [39, 54]. Another underlying theory emphasised enhancing patients’ recovery after surgery through various interventions and support systems designed to minimise risk factors for postoperative cognitive decline [39] and improving health and functional outcomes [53], which could subsequently lower the incidence of dNCR and p-NCD. Additional theoretical assumptions described the interventions as supporting patients’ psychological and emotional well-being, thereby promoting cognitive functioning [55] and minimising risk factors for cognitive decline [56].

##### Physical activity

The authors of physical activity studies [42, 57] suggest that physical activity, especially resistance training, can reduce systemic inflammation and support brain health in various ways. This is achieved partly by modulating neurotrophic factors, which are crucial for the growth and survival of neurons. Additionally, resistance training enhances neuroplasticity, allowing the brain to adapt and promote neurogenesis —the formation of new neurons. It also increases cerebral blood flow and enhances coordination between different brain regions.

## Discussion

This scoping review synthesised the literature on non-pharmacological interventions to support postoperative cognitive recovery after hospital discharge. The rationale for focusing on non-pharmacological interventions was to provide a perioperative care perspective. Pharmacological approaches have primarily targeted postoperative delirium rather than delayed neurocognitive recovery (dNCR) or postoperative neurocognitive disorder (p-NCD). Although dexmedetomidine has shown potential benefits in a recent meta-analysis, particularly for delirium, the certainty of the evidence remains very low [58].

The scoping review examined intervention types, theoretical frameworks, target populations, timing and duration, cognitive outcome measures, and reported findings. Twenty-one studies were identified and grouped into three categories: cognitive training, multimodal programs, and physical activity, with considerable variation in timing, delivery, and follow-up. While some interventions showed promise, findings were inconsistent and difficult to integrate due to methodological heterogeneity.

A central finding was the considerable variation in how interventions were conceptualised, delivered, and assessed. For example, six of the cognitive training interventions employed CCT [38, 43, 46, 48–50]. Compared to analogue training, CCT offers advantages such as automated difficulty adjustment, standardised delivery, and immediate performance feedback [59]. However, evidence suggests that CCT may not be the most effective complementary treatment for individuals with mild cognitive impairment (MCI) or dementia [60]. Among these six studies, one study utilised a mobile application featuring three distinct games designed to stimulate and engage different cognitive processes [49]. Notably, mobile gaming interventions have been reported to rank highest for improving global cognition and executive function, indicating potential benefits particularly for individuals with MCI [60]. Nevertheless, a Cochrane review published in 2019 concluded that the available evidence remains insufficient to determine whether CCT can improve or maintain cognitive function in individuals with existing cognitive impairment [61].

The duration and structure of the interventions differed, such as in cognitive training, which ranged from a single 90-minute preoperative session [40] to programmes extending 12 weeks postoperatively, with total exposure varying from 140 [41] to 2,160 min [38]. Similarly, multimodal interventions ranged from structured programs such as the Hospital Elder Life Program to various interdisciplinary approaches delivered during hospitalisation, as well as extended family-involved programs lasting several months after discharge. These multimodal interventions included team-based treatment planning, comprehensive geriatric assessments, orientation to time and space, therapeutic activities, early mobilisation, optimisation of pre- and postoperative nutrition, planning for early discharge support, and strategies to promote sleep [39, 51–54]. However, the considerable variation in both structure and scope across these interventions makes it difficult to compare outcomes. Importantly, interventions need to extend beyond the hospital environment and continue after discharge. Patients and their family members should not be left to manage the challenges of postoperative cognitive recovery alone. Consequently, it is crucial to provide adequate follow-up care and support for both patients and their families [62].

Outcome measures varied significantly among studies. Most studies utilised neurocognitive test batteries (*n* = 13), while some employed one or two screening tools (*n* = 8), with the MMSE being the most used. The MMSE is the most widely used screening tool in clinical settings and demonstrates moderate sensitivity and specificity for detecting dementia. However, its effectiveness in identifying mild p-NCD is limited [63], and it may have limited sensitivity and re-test reliability for detecting subtle changes over time. A more sensitive test is MoCA [64], which was used as an outcome measure in two studies that did not utilise full neurocognitive test batteries. In a 1995 Statement of Consensus, Murkin and colleagues emphasised that studies assessing postoperative cognitive dysfunction, i.e. dNCR and p-NCD, should utilise a neurocognitive test battery. Additionally, these studies should include at least one cognitive follow-up assessment when cognitive function has stabilised, ideally no earlier than one month after the surgery [65]. The use of less sensitive screening tools in several studies may therefore have contributed to the limited detection of intervention effects. The timing of postoperative evaluations also plays a key role in determining how frequently neurocognitive decline is identified [62]. In the present scoping review, the timing of assessments ranged from 24 h after surgery to 12 months. Most studies included three follow-up points, whereas others measured outcomes only once, making comparisons challenging. Furthermore, when planning neurocognitive assessments, it is important to balance the aim of evaluating multiple cognitive domains with practical constraints. For example, if a patient is experiencing anxiety related to upcoming surgery or has physical limitations, the test battery should not be overly demanding or lengthy, as prolonged assessments may lead to reduced performance due to pain, fatigue, or loss of engagement [62]. The findings of this scoping review may have been influenced by these considerations.

Most interventions in this review lacked explicit theoretical grounding. Among those that did, the explanations were predominantly biomedical, focusing on neuroplasticity, cerebral perfusion [36, 45], or cognitive reserve as mechanisms of benefit [46, 48, 49]. For example, authors suggested that cognitive training may stimulate prefrontal and parietal cortical activity or increase cognitive reserve, whereas physical activity studies emphasised modulation of inflammation and neurotrophic pathways. Conversely, only a minority linked outcomes to psychosocial or person-centred principles, for instance, multimodal interventions framed within emotional and social support [55]. This limited theoretical grounding restricts understanding of *how* and *for whom* interventions work, which is a key feature of complex interventions [66]. Although the mechanisms underlying postoperative neurocognitive decline remain incompletely understood, they are widely described as multifactorial. Among the proposed explanations, the neuroinflammation hypothesis is the most frequently cited [67–69]. These insights underscore the need for future research to integrate biomedical and psychosocial theories to explain intervention effects better and guide the development of comprehensive, person-centred strategies.

Only four of the 21 studies demonstrated clear effects on dNCR or p-NCD [35–37, 42, 49, 54]: two cognitive training trials [35–37, 49], one with physical activity as the intervention [42] and one multimodal intervention [54]. Nine studies did not directly assess dNCR or p-NCD; instead, they measured related constructs such as cognitive function, cognitive performance, or working memory. Several studies reported improvements in both intervention and control groups, or declines in both, suggesting either natural recovery patterns or intervention mechanisms insufficient to alter trajectories. Notably, cognitive training may introduce challenges such as feelings of being overwhelmed, anxiety, technological difficulties, and scheduling constraints prior to surgery, which can heighten anxiety and nervousness—particularly among older adults [70]. These findings underscore the need for interventions that are patient-centred, technologically accessible, and appropriately timed to minimise stress and maximise engagement.

Subjective cognitive complaints, which are a major concern for patients [19, 20] were rarely addressed in the included studies. Cognitive complaints include memory loss, inability to function, poor concentration, problems with social activities and activities of daily living, and fatigue [19, 20] and can affect daily tasks such as managing medication, preparing meals and remembering to eat [20], appointments and doing housework [19]. These are all important functions to maintain autonomy and self-capacity. A change in cognitive function may also affect the interaction and roles between the patient and their family, causing a sense of loss and frustration, especially among the patient’s next of kin. Only one study in this scoping review measured self-assessed cognitive failure and next-of-kin reports, and no benefit was observed. It has been noted that findings from neurocognitive tests are related to assessments made by close relatives [71] and that assessments by a next of kin are considered more reliable than patients’ self-assessment [72]. However, the assessment of next of kin regarding their family members’ cognitive symptoms, particularly regarding dNCR/p-NCD, is rarely studied [62]. This gap underscores the need to integrate patient-reported outcomes, as lived experiences frequently diverge from objective test results. According to the fifth edition of the *Diagnostic and Statistical Manual of Mental Disorders* (DSM-5) [73], diagnosing dNCR/pNCD requires subjective reports of cognitive difficulties from the patient, a caregiver, or a clinician, combined with objective neurocognitive data and evidence of decline in instrumental activities of daily living (IADL) [2, 10].

### Limitations

This scoping review has several limitations. First, grey literature and unpublished studies were not included, which may have introduced selection bias. Second, the categorisation of data may have been influenced by the researchers’ backgrounds, despite adherence to a structured data extraction and analysis protocol aligned with the research questions. Third, the review focused on studies published in English, which may limit the generalisability of findings and exclude relevant evidence from non-English sources. Finally, this study is inherent to the scoping review methodology. A scoping review does not aim to produce a critically appraised or synthesised response to a specific research question; rather, its purpose is to provide a broad overview or mapping of the available evidence [27]. Consequently, no formal assessment of methodological limitations or risk of bias was conducted for the included studies. Unlike systematic reviews, which typically exclude studies lacking power analyses or those with very small sample sizes, such as pilot and feasibility studies, the purpose of a scoping review is to explore how research is conducted within a specific field and to identify and analyse knowledge gaps [27]. This also means that the findings should be interpreted bearing this in mind, as the quality of the included studies was not evaluated.

## Conclusion

This scoping review indicates that current evidence is insufficient to support interventions that improve postoperative cognitive recovery; the evidence base remains fragmented and lacks strong theoretical grounding. Advancing the field will require an integrated approach that combines rigorous clinical trials, theoretical development, and implementation research. Future studies should prioritise standardised outcome measures, robust conceptual frameworks, and larger, well-designed trials to strengthen the evidence base and guide clinical practice.

## Supplementary Information

Below is the link to the electronic supplementary material.

Supplementary Material 1

## Data Availability

All data generated or analysed during this study are included in this published article and its supplementary information files.

## Abbreviations

BDNF: Brain-Derived Neurotrophic Factor
CNS: Central nervous system
CCT: Computerized cognitive training
CAM: Confusion Assessment Method
dNCR: Delayed neurocognitive recovery
DSM-5: Diagnostic and Statistical Manual of Mental Disorders
IADL: Instrumental activities of daily living
MoCA: Montreal Cognitive Assessment
NSE: Neuron-Specific Enolase
POCD: Postoperative cognitive decline
p-NCD: Postoperative neurocognitive disorder
QoL: Quality of Life
MMSE: The Mini-Mental State Examination
PRISMA-ScR: The Preferred Reporting Items for Systematic reviews and Meta-Analyses extension for Scoping Reviews
