# Supplementary material for: Interventions supporting cognitive recovery after surgery: a scoping review
Source: BMC Nurs. 2026 Apr 3;25:340. doi: 10.1186/s12912-026-04614-y (PMC13063448; doi:10.1186/s12912-026-04614-y)
Supplement: Supplementary file 1 — Supplementary Material 1 [file 12912_2026_4614_MOESM1_ESM.docx]

**Supplementary Table S1. Full search strategies for all databases** 1.Medline

| Interface: **Ovid MEDLINE(R) ALL** content coverage from 1946  Date of Search: 4 February 2025  Number of hits: 3,055  Comment: In Ovid, two or more words are automatically searched as phrases; i.e. no quotation marks are needed | Field labels   - exp/ = exploded MeSH term - / = non exploded MeSH term - .ti,ab,kf. = title, abstract and author keywords - adjx = within x words, regardless of order - * = truncation of word for alternate endings - ? = 0-1 letter/number - # = 1 letter/number |
| --- | --- |
| Database(s): **Ovid MEDLINE(R) ALL**1946 to February 03, 2025 Search Strategy:   \| **#** \| **Searches** \| **Results** \| \| --- \| --- \| --- \| \| 1 \| Postoperative Cognitive Complications/ \| 701 \| \| 2 \| Cognition/ or Cognition Disorders/ or Cognitive Dysfunction/ or Cognitive Dysfunction/ \| 230724 \| \| 3 \| exp Perioperative Care/ or exp Perioperative Period/ or Postoperative Complications/ \| 638219 \| \| 4 \| 2 and 3 \| 2940 \| \| 5 \| ((discharg* or postdischarg* or intraoperati* or intra-op* or perioperati* or peri-op* or postoperati* or post-op* or preoperati* or pre-op* or surgeries or surgery or surgical) adj (cogniti* or neurocogniti*)).ab. \| 3885 \| \| 6 \| ((discharg* or postdischarg* or intraoperati* or intra-op* or perioperati* or peri-op* or postoperati* or post-op* or preoperati* or pre-op* or surgeries or surgery or surgical) and (cogniti* or neurocogniti*)).ti. \| 4099 \| \| 7 \| ((discharg* or postdischarg* or intraoperati* or intra-op* or perioperati* or peri-op* or postoperati* or post-op* or preoperati* or pre-op* or surgeries or surgery or surgical) and (cogniti* or neurocogniti*)).kf. \| 2485 \| \| 8 \| ((discharg* or postdischarg* or intraoperati* or intra-op* or perioperati* or peri-op* or postoperati* or post-op* or preoperati* or pre-op* or surgeries or surgery or surgical) and brain health*).ti,ab,kf. \| 98 \| \| 9 \| ((after* or befor* or follow*) adj3 (operat* or surger*) adj3 (cogniti* or neurocogniti*)).ab. \| 1030 \| \| 10 \| ((after* or befor* or follow*) and (operat* or surger*) and (cogniti* or neurocogniti*)).ti. \| 949 \| \| 11 \| ((after* or befor* or follow*) and (operat* or surger*) and (cogniti* or neurocogniti*)).kf. \| 16 \| \| 12 \| (neurocogniti* recover* or dNCR or POCD or p-NCD or pNCD).ti,ab,kf. \| 1765 \| \| 13 \| or/1,4-12 \| 8553 \| \| 14 \| (randomized controlled trial or controlled clinical trial).pt. or randomi#ed.ti,ab. or placebo.ti,ab. or drug therapy.fs. or therapy.fs. or randomly.ti,ab. or trial.ti,ab. or groups.ti,ab. \| 8062330 \| \| 15 \| exp animals/ not humans.sh. \| 5304461 \| \| 16 \| 14 not 15 \| 7228690 \| \| 17 \| 13 and 16 \| 3055 \| | |

2. Psycinfo

| Interface: **EBSCOhost** - conctent coverage from 1806  Date of Search: 4 February 2025  Number of hits: 582 | Field labels   - DE = subject heading - TI = title - AB = abstract - KW = author keywords - Nx = within x words, regardless of order - * = truncation of word for alternate endings - # = 0-1 letter/number - ? = 1 letter/number - **Expanders** - Apply equivalent subjects - **Search modes** - Find all my search terms   Note: sometimes “quotation marks” are needed for single search terms to avoid automatic term mapping (lemmatization) |
| --- | --- |
| \| # \| Query \| Results \| \| --- \| --- \| --- \| \| S1 \| DE "Cognition" OR DE "Neurocognition" OR DE "Cognitive Impairment" \| 137,615 \| \| S2 \| DE "Postsurgical Complications" OR DE "Surgery" \| 16,547 \| \| S3 \| S1 AND S2 \| 602 \| \| S4 \| AB (((discharg* or postdischarg* or intraoperati* or intra-op* or perioperati* or peri-op* or postoperati* or post-op* or preoperati* or pre-op* or surgeries or surgery or surgical) W1 ((cogniti* or neurocogniti*))) \| 841 \| \| S5 \| ( TI (((discharg* or postdischarg* or intraoperati* or intra-op* or perioperati* or peri-op* or postoperati* or post-op* or preoperati* or pre-op* or surgeries or surgery or surgical) and (cogniti* or neurocogniti*))) ) OR KW ( (((discharg* or postdischarg* or intraoperati* or intra-op* or perioperati* or peri-op* or postoperati* or post-op* or preoperati* or pre-op* or surgeries or surgery or surgical) and (cogniti* or neurocogniti*))) ) \| 1,518 \| \| S6 \| TI ( ((discharg* or postdischarg* or intraoperati* or intra-op* or perioperati* or peri-op* or postoperati* or post-op* or preoperati* or pre-op* or surgeries or surgery or surgical) and "brain health*") ) OR KW ( ((discharg* or postdischarg* or intraoperati* or intra-op* or perioperati* or peri-op* or postoperati* or post-op* or preoperati* or pre-op* or surgeries or surgery or surgical) and "brain health*") ) OR AB ( ((discharg* or postdischarg* or intraoperati* or intra-op* or perioperati* or peri-op* or postoperati* or post-op* or preoperati* or pre-op* or surgeries or surgery or surgical) and "brain health*") ) \| 24 \| \| S7 \| TI ( ((after* or befor* or follow*) AND (operat* or surger*) AND (cogniti* or neurocogniti*)) ) OR KW ( ((after* or befor* or follow*) AND (operat* or surger*) AND (cogniti* or neurocogniti*)) ) OR AB ( ((after* or befor* or follow*) N2 (operat* or surger*) N2 (cogniti* or neurocogniti*)) ) \| 471 \| \| S8 \| TI ( ("neurocogniti* recover*" OR dNCR OR POCD OR p-NCD OR pNCD) ) OR KW ( ("neurocogniti* recover*" OR dNCR OR POCD OR p-NCD OR pNCD) ) OR AB ( ("neurocogniti* recover*" OR dNCR OR POCD OR p-NCD OR pNCD) ) \| 333 \| \| S9 \| S3 OR S4 OR S5 OR S6 OR S7 OR S8 \| 2,187 \| \| S10 \| MR "Treatment Outcome" OR MR "Clinical Trial" OR DE "Clinical Trials" OR DE Placebo \| 80,250 \| \| S11 \| TX ( "randomi?ed" OR "placebo" OR "randomly" OR "trial" OR "groups" ) OR ( TX((singl* OR doubl* OR treb* OR tripl*) W2 (blind* OR mask*)) ) OR ( TX((singl* OR doubl* OR tripl* OR trebl*) N3 (blind* OR dumm* OR mask*)) OR TX(clinical W2 trial*) ) \| 936,031 \| \| S12 \| S10 OR S11 \| 943,782 \| \| S13 \| S9 AND S12 \| 582 \| | |

3. Cinahl

| Interface: **EBSCOhost** - content coverage from 1981  Date of Search: 4 February 2025  Number of hits: 1,329 | Field labels   - MH+ = exploded Cinahl Heading - MH = non exploded Cinahl Heading - TI = title - AB = abstract - Nx = within x words, regardless of order - * = truncation of word for alternate endings - # = 0-1 letter/number - ? = 1 letter/number - **Expanders** - Apply equivalent subjects - **Search modes** - Find all my search terms   Note: sometimes “quotation marks” are needed for single search terms to avoid automatic term mapping (lemmatization) |
| --- | --- |
| \| # \| Query \| Results \| \| --- \| --- \| --- \| \| S1 \| (MH "Cognition") OR (MH "Cognition Disorders") OR (MH "Mild Cognitive Impairment") \| 105,941 \| \| S2 \| (MH "Perioperative Care+") OR (MH "Postoperative Period") OR (MH "Intraoperative Period") OR (MH "Preoperative Period") OR (MH "Postoperative Complications") \| 163,987 \| \| S3 \| S1 AND S2 \| 1,549 \| \| S4 \| TI (((discharg* or postdischarg* or intraoperati* or intra-op* or perioperati* or peri-op* or postoperati* or post-op* or preoperati* or pre-op* or surgeries or surgery or surgical) and (cogniti* or neurocogniti*))) \| 1,562 \| \| S5 \| AB (((discharg* or postdischarg* or intraoperati* or intra-op* or perioperati* or peri-op* or postoperati* or post-op* or preoperati* or pre-op* or surgeries or surgery or surgical) W1 ((cogniti* or neurocogniti*))) \| 1,078 \| \| S6 \| TI ( ((discharg* or postdischarg* or intraoperati* or intra-op* or perioperati* or peri-op* or postoperati* or post-op* or preoperati* or pre-op* or surgeries or surgery or surgical) and "brain health*") ) OR AB ( ((discharg* or postdischarg* or intraoperati* or intra-op* or perioperati* or peri-op* or postoperati* or post-op* or preoperati* or pre-op* or surgeries or surgery or surgical) and "brain health*") ) \| 28 \| \| S7 \| TI ( ((after* or befor* or follow*) AND (operat* or surger*) AND (cogniti* or neurocogniti*)) ) OR AB ( ((after* or befor* or follow*) N2 (operat* or surger*) N2 (cogniti* or neurocogniti*)) ) \| 552 \| \| S8 \| TI ( ("neurocogniti* recover*" or dNCR or POCD or p-NCD or pNCD) ) OR AB ( ("neurocogniti* recover*" or dNCR or POCD or p-NCD or pNCD) ) \| 390 \| \| S9 \| S3 OR S4 OR S5 OR S6 OR S7 OR S8 \| 3,118 \| \| S10 \| PT "Clinical trial" \| 114,106 \| \| S11 \| MH "Randomized Controlled Trials+" OR MH "Double-Blind Studies" OR MH "Single-Blind Studies" OR MH "Random Assignment" OR MH "Pretest-Posttest Design+" OR MH "Cluster Sample" OR MH "Sample Size" OR MH "Crossover Design" OR MH "Comparative Studies" OR MH "Placebos" OR PT (Randomized Controlled Trial) OR MW "Therapy" OR TI (randomised OR randomized) OR AB (random*) OR TI (trial) OR AB (trial) OR AB (assigned OR allocated OR control) OR AB (control W5 group) OR AB (cluster W3 rct) \| 2,549,357 \| \| S12 \| S10 OR S11 \| 2,561,755 \| \| S13 \| S9 AND S12 \| 1,329 \| | |

1. Medline

| Interface: **Ovid MEDLINE(R) ALL** content coverage from 1946 to April 15, 2025  Date of Search: 16 April 2025  Number of hits: 3,278  Comment: In Ovid, two or more words are automatically searched as phrases; i.e. no quotation marks are needed | Field labels   - exp/ = exploded MeSH term - / = non exploded MeSH term - .ti,ab,kf. = title, abstract and author keywords - adjx = within x words, regardless of order - * = truncation of word for alternate endings - ? = 0-1 letter/number - # = 1 letter/number |
| --- | --- |
| \| **#** \| **Searches** \| **Results** \| \| --- \| --- \| --- \| \| 1 \| Postoperative Cognitive Complications/ \| 743 \| \| 2 \| Cognition/ or Cognition Disorders/ or Cognitive Dysfunction/ or Cognitive Dysfunction/ \| 233141 \| \| 3 \| exp Perioperative Care/ or exp Perioperative Period/ or Postoperative Complications/ \| 642338 \| \| 4 \| 2 and 3 \| 2957 \| \| 5 \| ((discharg* or postdischarg* or intraoperati* or intra-op* or perioperati* or peri-op* or postoperati* or post-op* or preoperati* or pre-op* or surgeries or surgery or surgical) adj (cogniti* or neurocogniti*)).ab. \| 3977 \| \| 6 \| ((discharg* or postdischarg* or intraoperati* or intra-op* or perioperati* or peri-op* or postoperati* or post-op* or preoperati* or pre-op* or surgeries or surgery or surgical) and (cogniti* or neurocogniti*)).kf. \| 2561 \| \| 7 \| ((discharg* or postdischarg* or intraoperati* or intra-op* or perioperati* or peri-op* or postoperati* or post-op* or preoperati* or pre-op* or surgeries or surgery or surgical) and brain health*).ti,ab,kf. \| 100 \| \| 8 \| ((discharg* or postdischarg* or intraoperati* or intra-op* or perioperati* or peri-op* or postoperati* or post-op* or preoperati* or pre-op* or surgeries or surgery or surgical) and (cogniti* or neurocogniti*)).ti. \| 4184 \| \| 9 \| ((after* or befor* or follow*) adj3 (operat* or surger*) adj3 (cogniti* or neurocogniti*)).ab. \| 1047 \| \| 10 \| ((after* or befor* or follow*) and (operat* or surger*) and (cogniti* or neurocogniti*)).ti. \| 963 \| \| 11 \| ((after* or befor* or follow*) and (operat* or surger*) and (cogniti* or neurocogniti*)).kf. \| 16 \| \| 12 \| (neurocogniti* recover* or dNCR or POCD or p-NCD or pNCD).ti,ab,kf. \| 1813 \| \| 13 \| or/1,4-12 \| 8701 \| \| 14 \| (randomized controlled trial or controlled clinical trial).pt. or randomi#ed.ti,ab. or placebo.ti,ab. or drug therapy.fs. or therapy.fs. or randomly.ti,ab. or trial.ti,ab. or groups.ti,ab. \| 8133144 \| \| 15 \| exp animals/ not humans.sh. \| 5328140 \| \| 16 \| 14 not 15 \| 7292791 \| \| 17 \| Clinical Protocols/ or "Clinical Trial Protocols as Topic"/ or Clinical Trial Protocol.pt. or Feasibility Studies/ or Pilot Projects/ or Preliminary Data/ \| 284440 \| \| 18 \| (feasibility or pilot).ti,ab. \| 476020 \| \| 19 \| (preliminar* adj3 (data or result*)).ti,ab. \| 85771 \| \| 20 \| ((clinical or treatment* or trial or research*) and protocol*).ti. \| 37228 \| \| 21 \| ((clinical or treatment* or trial or research*) adj3 protocol*).ab. \| 56735 \| \| 22 \| or/17-21 \| 720953 \| \| 23 \| 22 not 15 \| 675945 \| \| 24 \| 16 or 23 \| 7682091 \| \| 25 \| 13 and 24 \| 3278 \| | |

2. Psycinfo

| Interface: **EBSCOhost** - conctent coverage from 1806  Date of Search: 16 April 2025  Number of hits: 632 | Field labels   - DE = subject heading - TI = title - AB = abstract - KW = author keywords - Nx = within x words, regardless of order - * = truncation of word for alternate endings - # = 0-1 letter/number - ? = 1 letter/number - **Expanders** - Apply equivalent subjects - **Search modes** - Find all my search terms   Note: sometimes “quotation marks” are needed for single search terms to avoid automatic term mapping (lemmatization) |
| --- | --- |
| \| # \| Query \| Results \| \| --- \| --- \| --- \| \| S1 \| DE "Cognition" OR DE "Neurocognition" OR DE "Cognitive Impairment" \| 138,454 \| \| S2 \| DE "Postsurgical Complications" OR DE "Surgery" \| 16,617 \| \| S3 \| S1 AND S2 \| 611 \| \| S4 \| AB (((discharg* or postdischarg* or intraoperati* or intra-op* or perioperati* or peri-op* or postoperati* or post-op* or preoperati* or pre-op* or surgeries or surgery or surgical) W1 ((cogniti* or neurocogniti*))) \| 855 \| \| S5 \| ( TI (((discharg* or postdischarg* or intraoperati* or intra-op* or perioperati* or peri-op* or postoperati* or post-op* or preoperati* or pre-op* or surgeries or surgery or surgical) and (cogniti* or neurocogniti*))) ) OR KW ( (((discharg* or postdischarg* or intraoperati* or intra-op* or perioperati* or peri-op* or postoperati* or post-op* or preoperati* or pre-op* or surgeries or surgery or surgical) and (cogniti* or neurocogniti*))) ) \| 1,526 \| \| S6 \| TI ( ((discharg* or postdischarg* or intraoperati* or intra-op* or perioperati* or peri-op* or postoperati* or post-op* or preoperati* or pre-op* or surgeries or surgery or surgical) and "brain health*") ) OR KW ( ((discharg* or postdischarg* or intraoperati* or intra-op* or perioperati* or peri-op* or postoperati* or post-op* or preoperati* or pre-op* or surgeries or surgery or surgical) and "brain health*") ) OR AB ( ((discharg* or postdischarg* or intraoperati* or intra-op* or perioperati* or peri-op* or postoperati* or post-op* or preoperati* or pre-op* or surgeries or surgery or surgical) and "brain health*") ) \| 25 \| \| S7 \| TI ( ((after* or befor* or follow*) AND (operat* or surger*) AND (cogniti* or neurocogniti*)) ) OR KW ( ((after* or befor* or follow*) AND (operat* or surger*) AND (cogniti* or neurocogniti*)) ) OR AB ( ((after* or befor* or follow*) N2 (operat* or surger*) N2 (cogniti* or neurocogniti*)) ) \| 476 \| \| S8 \| TI ( ("neurocogniti* recover*" OR dNCR OR POCD OR p-NCD OR pNCD) ) OR KW ( ("neurocogniti* recover*" OR dNCR OR POCD OR p-NCD OR pNCD) ) OR AB ( ("neurocogniti* recover*" OR dNCR OR POCD OR p-NCD OR pNCD) ) \| 340 \| \| S9 \| S3 OR S4 OR S5 OR S6 OR S7 OR S8 \| 2,197 \| \| S10 \| MR "Treatment Outcome" OR MR "Clinical Trial" OR DE "Clinical Trials" OR DE Placebo \| 81,266 \| \| S11 \| TX ( "randomi?ed" OR "placebo" OR "randomly" OR "trial" OR "groups" ) OR ( TX((singl* OR doubl* OR treb* OR tripl*) W2 (blind* OR mask*)) ) OR ( TX((singl* OR doubl* OR tripl* OR trebl*) N3 (blind* OR dumm* OR mask*)) OR TX(clinical W2 trial*) ) \| 943,725 \| \| S12 \| S10 OR S11 \| 951,533 \| \| S13 \| XB (feasibility OR pilot) \| 87,962 \| \| S14 \| XB ((preliminar* N2 (data OR result*))) \| 16,087 \| \| S15 \| TI (((clinical OR treatment* OR trial OR research*) AND protocol*)) \| 2,591 \| \| S16 \| AB (((clinical OR treatment* OR trial OR research*) N2 protocol*)) \| 8,134 \| \| S17 \| S13 OR S14 OR S15 OR S16 \| 110,823 \| \| S18 \| S12 OR S17 \| 1,023,558 \| \| S19 \| S9 AND S18 \| 632 \| | |

3. Cinahl

| Interface: **EBSCOhost** - content coverage from 1981  Date of Search: 16 April 2025  Number of hits: 1,404 | Field labels   - MH+ = exploded Cinahl Heading - MH = non exploded Cinahl Heading - TI = title - AB = abstract - Nx = within x words, regardless of order - * = truncation of word for alternate endings - # = 0-1 letter/number - ? = 1 letter/number - **Expanders** - Apply equivalent subjects - **Search modes** - Find all my search terms   Note: sometimes “quotation marks” are needed for single search terms to avoid automatic term mapping (lemmatization) |
| --- | --- |
| \| # \| Query \| Results \| \| --- \| --- \| --- \| \| S1 \| (MH "Cognition") OR (MH "Cognition Disorders") OR (MH "Mild Cognitive Impairment") \| 107,326 \| \| S2 \| (MH "Perioperative Care+") OR (MH "Postoperative Period") OR (MH "Intraoperative Period") OR (MH "Preoperative Period") OR (MH "Postoperative Complications") \| 165,665 \| \| S3 \| S1 AND S2 \| 1,567 \| \| S4 \| TI (((discharg* or postdischarg* or intraoperati* or intra-op* or perioperati* or peri-op* or postoperati* or post-op* or preoperati* or pre-op* or surgeries or surgery or surgical) and (cogniti* or neurocogniti*))) \| 1,590 \| \| S5 \| AB (((discharg* or postdischarg* or intraoperati* or intra-op* or perioperati* or peri-op* or postoperati* or post-op* or preoperati* or pre-op* or surgeries or surgery or surgical) W1 ((cogniti* or neurocogniti*))) \| 1,097 \| \| S6 \| TI ( ((discharg* or postdischarg* or intraoperati* or intra-op* or perioperati* or peri-op* or postoperati* or post-op* or preoperati* or pre-op* or surgeries or surgery or surgical) and "brain health*") ) OR AB ( ((discharg* or postdischarg* or intraoperati* or intra-op* or perioperati* or peri-op* or postoperati* or post-op* or preoperati* or pre-op* or surgeries or surgery or surgical) and "brain health*") ) \| 29 \| \| S7 \| TI ( ((after* or befor* or follow*) AND (operat* or surger*) AND (cogniti* or neurocogniti*)) ) OR AB ( ((after* or befor* or follow*) N2 (operat* or surger*) N2 (cogniti* or neurocogniti*)) ) \| 559 \| \| S8 \| TI ( ("neurocogniti* recover*" or dNCR or POCD or p-NCD or pNCD) ) OR AB ( ("neurocogniti* recover*" or dNCR or POCD or p-NCD or pNCD) ) \| 400 \| \| S9 \| S3 OR S4 OR S5 OR S6 OR S7 OR S8 \| 3,161 \| \| S10 \| PT "Clinical trial" \| 114,563 \| \| S11 \| MH "Randomized Controlled Trials+" OR MH "Double-Blind Studies" OR MH "Single-Blind Studies" OR MH "Random Assignment" OR MH "Pretest-Posttest Design+" OR MH "Cluster Sample" OR MH "Sample Size" OR MH "Crossover Design" OR MH "Comparative Studies" OR MH "Placebos" OR PT (Randomized Controlled Trial) OR MW "Therapy" OR TI (randomised OR randomized) OR AB (random*) OR TI (trial) OR AB (trial) OR AB (assigned OR allocated OR control) OR AB (control W5 group) OR AB (cluster W3 rct) \| 2,571,660 \| \| S12 \| S10 OR S11 \| 2,584,115 \| \| S13 \| (MH "Pilot Studies") OR PT "Protocol" OR (MH "Protocols") OR (MH "Research Protocols") \| 136,834 \| \| S14 \| XB (feasibility OR pilot) \| 143,819 \| \| S15 \| XB ((preliminar* N2 (data OR result*))) \| 16,944 \| \| S16 \| TI (((clinical OR treatment* OR trial OR research*) AND protocol*)) \| 18,152 \| \| S17 \| AB (((clinical OR treatment* OR trial OR research*) N2 protocol*)) \| 15,296 \| \| S18 \| S13 OR S14 OR S15 OR S16 OR S17 \| 248,186 \| \| S19 \| S12 OR S18 \| 2,698,111 \| \| S20 \| S9 AND S19 \| 1,404 \| | |

1. Medline

| Interface: **Ovid MEDLINE(R) ALL** content coverage from 1946 to April 15, 2025  Date of Search: 17 October 2025  Number of hits: 3,472  Comment: In Ovid, two or more words are automatically searched as phrases; i.e. no quotation marks are needed | Field labels   - exp/ = exploded MeSH term - / = non exploded MeSH term - .ti,ab,kf. = title, abstract and author keywords - adjx = within x words, regardless of order - * = truncation of word for alternate endings - ? = 0-1 letter/number - # = 1 letter/number |
| --- | --- |
| Database(s): **Ovid MEDLINE(R) ALL**1946 to October 16, 2025 Search Strategy:   \| **#** \| **Searches** \| **Results** \| \| --- \| --- \| --- \| \| 1 \| Postoperative Cognitive Complications/ \| 833 \| \| 2 \| Cognition/ or Cognition Disorders/ or Cognitive Dysfunction/ \| 239230 \| \| 3 \| exp Perioperative Care/ or exp Perioperative Period/ or Postoperative Complications/ \| 653026 \| \| 4 \| 2 and 3 \| 2994 \| \| 5 \| ((discharg* or postdischarg* or intraoperati* or intra-op* or perioperati* or peri-op* or postoperati* or post-op* or preoperati* or pre-op* or surgeries or surgery or surgical) adj (cogniti* or neurocogniti*)).ab. \| 4256 \| \| 6 \| ((discharg* or postdischarg* or intraoperati* or intra-op* or perioperati* or peri-op* or postoperati* or post-op* or preoperati* or pre-op* or surgeries or surgery or surgical) and (cogniti* or neurocogniti*)).kf. \| 2826 \| \| 7 \| ((discharg* or postdischarg* or intraoperati* or intra-op* or perioperati* or peri-op* or postoperati* or post-op* or preoperati* or pre-op* or surgeries or surgery or surgical) and (cogniti* or neurocogniti*)).ti. \| 4458 \| \| 8 \| ((discharg* or postdischarg* or intraoperati* or intra-op* or perioperati* or peri-op* or postoperati* or post-op* or preoperati* or pre-op* or surgeries or surgery or surgical) and brain health*).ti,ab,kf. \| 128 \| \| 9 \| ((after* or befor* or follow*) adj3 (operat* or surger*) adj3 (cogniti* or neurocogniti*)).ab. \| 1088 \| \| 10 \| ((after* or befor* or follow*) and (operat* or surger*) and (cogniti* or neurocogniti*)).ti. \| 1007 \| \| 11 \| ((after* or befor* or follow*) and (operat* or surger*) and (cogniti* or neurocogniti*)).kf. \| 16 \| \| 12 \| (neurocogniti* recover* or dNCR or POCD or p-NCD or pNCD).ti,ab,kf. \| 1921 \| \| 13 \| or/1,4-12 \| 9211 \| \| 14 \| (randomized controlled trial or controlled clinical trial).pt. or randomi#ed.ti,ab. or placebo.ti,ab. or drug therapy.fs. or therapy.fs. or randomly.ti,ab. or trial.ti,ab. or groups.ti,ab. \| 8345993 \| \| 15 \| Clinical Protocols/ or "Clinical Trial Protocols as Topic"/ or Clinical Trial Protocol.pt. or Feasibility Studies/ or Pilot Projects/ or Preliminary Data/ \| 292499 \| \| 16 \| (feasibility or pilot).ti,ab. \| 497070 \| \| 17 \| (preliminar* adj3 (data or result*)).ti,ab. \| 87315 \| \| 18 \| ((clinical or treatment* or trial or research*) and protocol*).ti. \| 39571 \| \| 19 \| ((clinical or treatment* or trial or research*) adj3 protocol*).ab. \| 60182 \| \| 20 \| or/14-19 \| 8783938 \| \| 21 \| exp animals/ not humans.sh. \| 5385097 \| \| 22 \| 20 not 21 \| 7893865 \| \| 23 \| 13 and 22 \| 3472 \| | |

2. Psycinfo

| Interface: **EBSCOhost** - conctent coverage from 1806  Date of Search: 17 October 2025  Number of hits: 644 | Field labels   - DE = subject heading - TI = title - AB = abstract - KW = author keywords - Nx = within x words, regardless of order - * = truncation of word for alternate endings - # = 0-1 letter/number - ? = 1 letter/number - **Expanders** - Apply equivalent subjects - **Search modes** - Find all my search terms   Note: sometimes “quotation marks” are needed for single search terms to avoid automatic term mapping (lemmatization) |
| --- | --- |
| \| **S#** \| **Query (user-entered)** \| **Results (count)** \| \| --- \| --- \| --- \| \| S1 \| DE "Cognition" OR DE "Neurocognition" OR DE "Cognitive Impairment" \| 141151 \| \| S2 \| DE "Postsurgical Complications" OR DE "Surgery" \| 16966 \| \| S3 \| S1 AND S2 \| 632 \| \| S4 \| AB (((discharg* or postdischarg* or intraoperati* or intra-op* or perioperati* or peri-op* or postoperati* or post-op* or preoperati* or pre-op* or surgeries or surgery or surgical) W1 ((cogniti* or neurocogniti*))) \| 890 \| \| S5 \| ( TI (((discharg* or postdischarg* or intraoperati* or intra-op* or perioperati* or peri-op* or postoperati* or post-op* or preoperati* or pre-op* or surgeries or surgery or surgical) and (cogniti* or neurocogniti*))) ) OR KW ( (((discharg* or postdischarg* or intraoperati* or intra-op* or perioperati* or peri-op* or postoperati* or post-op* or preoperati* or pre-op* or surgeries or surgery or surgical) and (cogniti* or neurocogniti*))) ) \| 1578 \| \| S6 \| ( TI (((discharg* or postdischarg* or intraoperati* or intra-op* or perioperati* or peri-op* or postoperati* or post-op* or preoperati* or pre-op* or surgeries or surgery or surgical) and (cogniti* or neurocogniti*))) ) OR KW ( (((discharg* or postdischarg* or intraoperati* or intra-op* or perioperati* or peri-op* or postoperati* or post-op* or preoperati* or pre-op* or surgeries or surgery or surgical) and (cogniti* or neurocogniti*))) ) \| 1578 \| \| S7 \| TI ( ((after* or befor* or follow*) AND (operat* or surger*) AND (cogniti* or neurocogniti*)) ) OR KW ( ((after* or befor* or follow*) AND (operat* or surger*) AND (cogniti* or neurocogniti*)) ) OR AB ( ((after* or befor* or follow*) N2 (operat* or surger*) N2 (cogniti* or neurocogniti*)) ) \| 487 \| \| S8 \| TI ( ("neurocogniti* recover*" OR dNCR OR POCD OR p-NCD OR pNCD) ) OR KW ( ("neurocogniti* recover*" OR dNCR OR POCD OR p-NCD OR pNCD) ) OR AB ( ("neurocogniti* recover*" OR dNCR OR POCD OR p-NCD OR pNCD) ) \| 355 \| \| S9 \| S3 OR S4 OR S5 OR S6 OR S7 OR S8 \| 2249 \| \| S10 \| MR "Treatment Outcome" OR MR "Clinical Trial" OR DE "Clinical Trials" OR DE Placebo \| 84020 \| \| S11 \| TX ( "randomi?ed" OR "placebo" OR "randomly" OR "trial" OR "groups" ) OR ( TX((singl* OR doubl* OR treb* OR tripl*) W2 (blind* OR mask*)) ) OR ( TX((singl* OR doubl* OR tripl* OR trebl*) N3 (blind* OR dumm* OR mask*)) OR TX(clinical W2 trial*) ) \| 964925 \| \| S12 \| XB (feasibility OR pilot) \| 90640 \| \| S13 \| XB ((preliminar* N2 (data OR result*))) \| 16374 \| \| S14 \| TI (((clinical OR treatment* OR trial OR research*) AND protocol*)) \| 2769 \| \| S15 \| AB (((clinical OR treatment* OR trial OR research*) N2 protocol*)) \| 8368 \| \| S16 \| S10 OR S11 OR S12 OR S13 OR S14 OR S15 \| 1046309 \| \| S17 \| S9 AND S16 \| 644 \| | |

3. Cinahl

| Interface: **EBSCOhost** - content coverage from 1981  Date of Search: 17 October 2025  Number of hits: 1,442 | Field labels   - MH+ = exploded Cinahl Heading - MH = non exploded Cinahl Heading - TI = title - AB = abstract - Nx = within x words, regardless of order - * = truncation of word for alternate endings - # = 0-1 letter/number - ? = 1 letter/number - **Expanders** - Apply equivalent subjects - **Search modes** - Find all my search terms   Note: sometimes “quotation marks” are needed for single search terms to avoid automatic term mapping (lemmatization) |
| --- | --- |
| \| **S#** \| **Query (user-entered)** \| **Results (count)** \| \| --- \| --- \| --- \| \| S1 \| (MH "Cognition") OR (MH "Cognition Disorders") OR (MH "Mild Cognitive Impairment") \| 111456 \| \| S2 \| (MH "Perioperative Care+") OR (MH "Postoperative Period") OR (MH "Intraoperative Period") OR (MH "Preoperative Period") OR (MH "Postoperative Complications") \| 170507 \| \| S3 \| S1 AND S2 \| 1623 \| \| S4 \| TI (((discharg* or postdischarg* or intraoperati* or intra-op* or perioperati* or peri-op* or postoperati* or post-op* or preoperati* or pre-op* or surgeries or surgery or surgical) and (cogniti* or neurocogniti*))) \| 1656 \| \| S5 \| AB (((discharg* or postdischarg* or intraoperati* or intra-op* or perioperati* or peri-op* or postoperati* or post-op* or preoperati* or pre-op* or surgeries or surgery or surgical) W1 ((cogniti* or neurocogniti*))) \| 1112 \| \| S6 \| TI ( ((discharg* or postdischarg* or intraoperati* or intra-op* or perioperati* or peri-op* or postoperati* or post-op* or preoperati* or pre-op* or surgeries or surgery or surgical) and "brain health*") ) OR AB ( ((discharg* or postdischarg* or intraoperati* or intra-op* or perioperati* or peri-op* or postoperati* or post-op* or preoperati* or pre-op* or surgeries or surgery or surgical) and "brain health*") ) \| 31 \| \| S7 \| TI ( ((after* or befor* or follow*) AND (operat* or surger*) AND (cogniti* or neurocogniti*)) ) OR AB ( ((after* or befor* or follow*) N2 (operat* or surger*) N2 (cogniti* or neurocogniti*)) ) \| 578 \| \| S8 \| TI ( ("neurocogniti* recover*" or dNCR or POCD or p-NCD or pNCD) ) OR AB ( ("neurocogniti* recover*" or dNCR or POCD or p-NCD or pNCD) ) \| 408 \| \| S9 \| S3 OR S4 OR S5 OR S7 OR S8 \| 3259 \| \| S10 \| PT "Clinical trial" \| 116232 \| \| S11 \| MH "Randomized Controlled Trials+" OR MH "Double-Blind Studies" OR MH "Single-Blind Studies" OR MH "Random Assignment" OR MH "Pretest-Posttest Design+" OR MH "Cluster Sample" OR MH "Sample Size" OR MH "Crossover Design" OR MH "Comparative Studies" OR MH "Placebos" OR PT (Randomized Controlled Trial) OR MW "Therapy" OR TI (randomised OR randomized) OR AB (random*) OR TI (trial) OR AB (trial) OR AB (assigned OR allocated OR control) OR AB (control W5 group) OR AB (cluster W3 rct) \| 2630405 \| \| S12 \| (MH "Pilot Studies") OR PT "Protocol" OR (MH "Protocols") OR (MH "Research Protocols") \| 140866 \| \| S13 \| XB (feasibility OR pilot) \| 148480 \| \| S14 \| XB ((preliminar* N2 (data OR result*))) \| 17298 \| \| S15 \| TI (((clinical OR treatment* OR trial OR research*) AND protocol*)) \| 18769 \| \| S16 \| AB (((clinical OR treatment* OR trial OR research*) N2 protocol*)) \| 15790 \| \| S17 \| S10 OR S11 OR S12 OR S13 OR S14 OR S15 OR S16 \| 2760617 \| \| S18 \| S9 AND S17 \| 1442 \| | |
